# Maintained larval growth in mussel larvae exposed to acidified under-saturated seawater

**DOI:** 10.1038/srep23728

**Published:** 2016-03-29

**Authors:** Alexander Ventura, Sabrina Schulz, Sam Dupont

**Affiliations:** 1 Department of Biological and Environmental Sciences, University of Gothenburg, The Sven Lovén Centre for Marine Sciences, Kristineberg 566, SE-451 78 Fiskebäckskil, Sweden

## Abstract

Ocean acidification (OA) is known to affect bivalve early life-stages. We tested responses of blue mussel larvae to a wide range of pH in order to identify their tolerance threshold. Our results confirmed that decreasing seawater pH and decreasing saturation state increases larval mortality rate and the percentage of abnormally developing larvae. Virtually no larvae reared at average pH_T_ 7.16 were able to feed or reach the D-shell stage and their development appeared to be arrested at the trochophore stage. However larvae were capable of reaching the D-shell stage under milder acidification (pH_T_ ≈ 7.35, 7.6, 7.85) including in under-saturated seawater with Ω_*a*_ as low as 0.54 ± 0.01 (mean ± s. e. m.), with a tipping point for normal development identified at pH_T_ 7.765. Additionally growth rate of normally developing larvae was not affected by lower pH_T_ despite potential increased energy costs associated with compensatory calcification in response to increased shell dissolution. Overall, our results on OA impacts on mussel larvae suggest an average pH_T_ of 7.16 is beyond their physiological tolerance threshold and indicate a shift in energy allocation towards growth in some individuals revealing potential OA resilience.

Human induced rise in atmospheric CO_2_ concentration has been linked to changes in seawater carbonate chemistry and a decrease in ocean pH, a process known as ocean acidification (OA). This process is likely to have severe effects on marine species and ecosystems. An emblematic case is the OA induced mass mortality of larvae in oyster hatcheries on the West coast of the United States between 2005 and 2009. These events severely affected the highly lucrative oyster industry in the area with a loss of US$110 million^1^. For marine calcifying species what is sometimes considered as being one of the main drivers of responses to OA is calcium carbonate saturation state. In addition to a decrease in seawater pH, OA is in fact also characterized by a reduction in carbonate ion (CO_3_^2−^) concentration and saturation state (Ω) of the various forms of calcium carbonate (CaCO_3_) such as calcite (Ω_*c*_) and aragonite (Ω_*a*_) which from a strictly chemical perspective can make seawater corrosive to CaCO_3 _2. However responses to OA are species-specific and bio-calcification is a highly controlled physiological process^3^. Mount *et al.*^4^ showed that calcium carbonate production is at least partly intracellular in the eastern oyster *Crassostrea virginica* (Gmelin, 1791). Roleda *et al.*^3^ present several examples of marine organisms capable of controlling the carbonate chemistry of the fluid media at calcification sites. This ability allows calcifiers to adapt to extremely low pH and under-saturated habitats. An example are vent mussels capable of precipitating calcium carbonate at pH ranging between 5.36 and 7.29^5^. As a consequence we can predict that a species’ physiological tipping point will be related to its niche and natural environmental variability rather than simple chemical thresholds^6^,^7^.

The species investigated in this study was the blue mussel *Mytilus edulis* (Linnaeus, 1758) which inhabits shallow coastal environments, is eurythermal, with lower and upper thermal tolerance limits of −10° and 30 °C, respectively^8^,^9^, and tolerates wide fluctuations in salinity and oxygen tension^10^. Impacts of pH on *M. edulis* and their larvae have been widely investigated. Gazeau *et al.*^11^ demonstrated that calcification rates of blue mussels decrease linearly with short term exposure to increasing carbon dioxide partial pressure (*p*CO_2_). Thomsen and Melzner^12^ analysed the impacts of pH on metabolism in adult *M. edulis*. The authors observed reduced shell growth under severe acidification and suggested this may be a result of synergistic effects of increased cellular energy demand and nitrogen loss. A study on the effects of OA on early life stages of *M. edulis*^13^ showed negative impacts of increasing seawater acidity on parameters such as hatching rates and D-veliger shell growth. During a study conducted by Bechmann *et al.*^14^ early life stages of *M. edulis* were exposed to pH 7.6. No significant effect on fertilization success, development time, shell abnormality or feeding was observed as compared to pH 8.1. Mussels were still able to calcify in seawater under-saturated with respect to aragonite but were significantly smaller. Despite existing information on OA impacts on blue mussels, it is currently not known what the physiological thresholds of their sensitive larval stage may be. The aim of our study was to investigate the effects of a wide range of seawater pH on different physiological parameters of *M. edulis* developing larvae in order to find the physiological tipping point beyond which they are no longer capable of carrying out those functions necessary to their survival and recruitment into the adult population. We hypothesized that mussel larvae would be able to develop normally and grow under the pH range they naturally experience at collection site, between 8.7 and 7.6^15^.

## Results

A summary of seawater carbonate chemistry measurements is presented in Table 1. pH_T_ was stable and did not fluctuate more than 0.7% in any of the cultures throughout the experiment. There was a significant difference in pH_T_ between all nominal pH treatments (Kruskal-Wallis: chi-squared = 46.3895, df = 3, *p* = 4.687e^−10^). Calculated average *p*CO_2_ in cultures ranged between 334 ± 6 (nominal pH = 8.1, ambient conditions) and 3712 ± 160 (nominal pH = 7.1) μatm. All cultures with nominal pH ≤ 7.6 were under-saturated with respect to aragonite whilst calcite under-saturation was observed at nominal pH ≤ 7.35.

**Figure 1:**
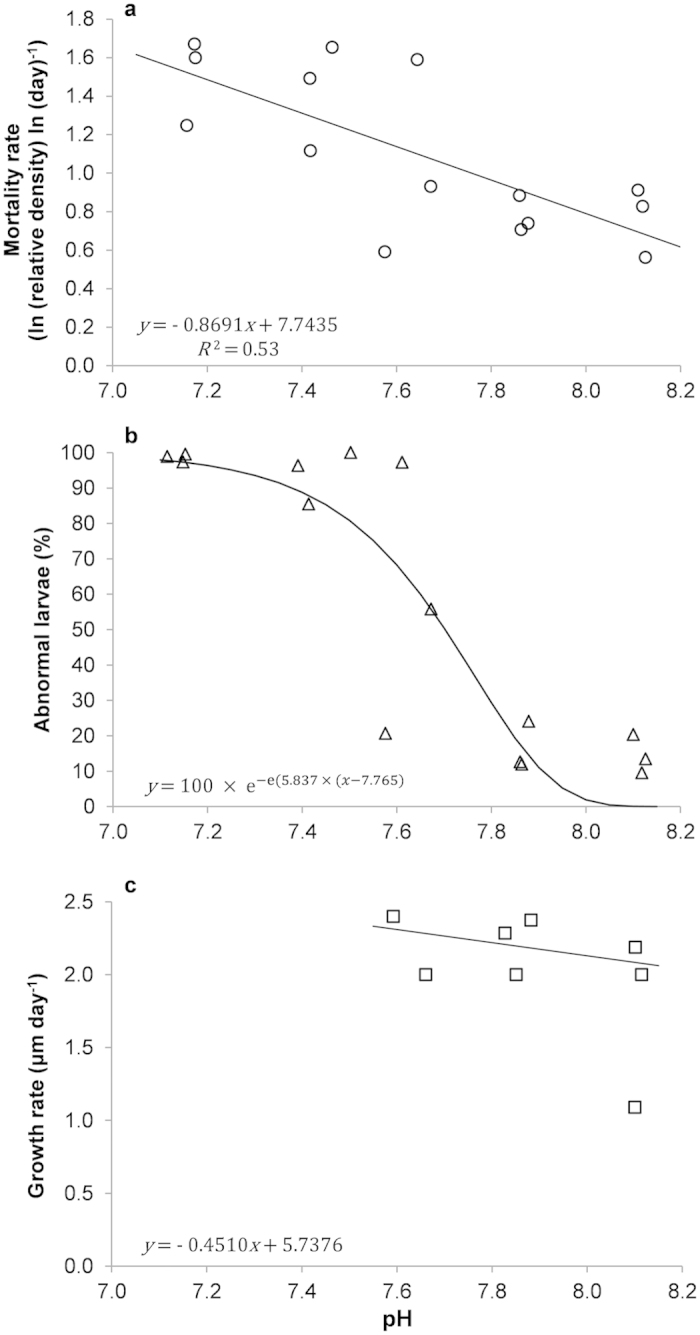
Decreasing mortality, abnormality and maintained growth rates with increasing pH. (**a**) Relationship between larval mortality rates (ln (relative density) ln (day)^−1^) and average pH_T_. Mortality rates for each point correspond to the regression slope coefficients extracted from significant power relationships between relative density and time (day). For clarity y axis values have been converted to positive values. (**b**) Relationship between the percentage of larval abnormality and average pH_T_. (**c**) Relationship between growth rate (μm day^−1^) of normally D-shaped larvae and average pH_T_. Growth rates for each point correspond to the regression slope coefficients extracted from significant Theil-Sen median based linear relationships between mean larval length (μm) and time (day).

Mortality rates significantly increased with decreasing average pH_T_ (Linear regression: F_1,13_ = 15.12; R^2^ = 0.53; *p* = 0.0018) (Fig. 1a). Larvae in replicates reared at nominal pH 7.1 did not survive beyond day 27–29; in one of the nominal pH 7.35 replicates no larvae survived beyond day 29 and all larvae in one of the nominal pH 7.6 replicates died after day 24.

**Figure 2:**
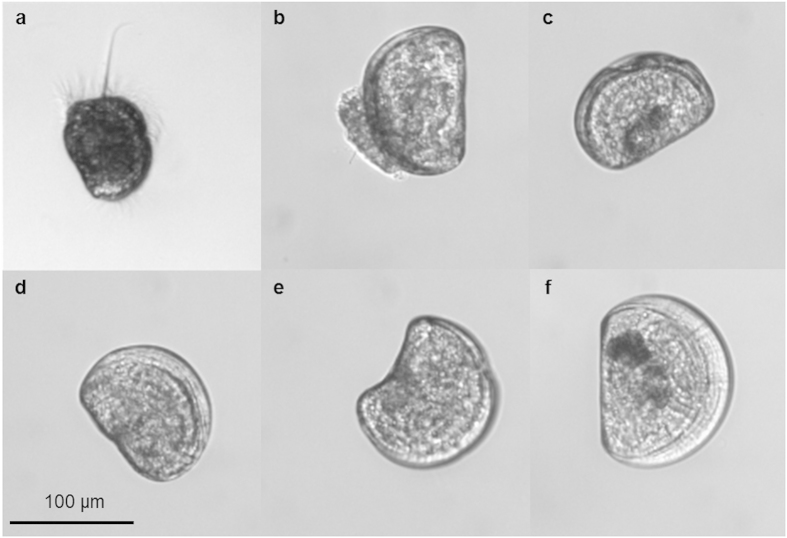
Most common larval phenotypes observed throughout the course of the experiment. (**a**) Trochophore stage, (**b**) protruding mantle, (**c**) indented margin, (**d**) convex hinge, (**e**) cupped and (**f**) normally D-shaped larva. N. B. All photos are from experiment day 11.

Percentage of abnormally developing mussel larvae (see Fig. 2a–e for abnormal phenotypes as compared to normal veliger D-shaped larvae, Fig. 2f) was significantly affected by pH_T_ (Gompertz model: F_2,13_ = 88.39; p < 0.0001) with the tipping point corresponding to pH = 7.765 (Fig. 1b). The percentage of abnormally D-shaped (Fig. 2b–e) and trochophore larvae (Fig. 2a) increased with decreasing pH_T_ (Table 2). Despite observation of very few D-shaped larvae almost all larvae within the lowest pH treatment (average pH = 7.16) were unable to reach the veliger stage and their development appeared to be arrested at the trochophore stage. A very low number of normally D-shaped larvae were observed at nominal pH 7.35.

Growth of normally D-shaped larvae was not significantly affected by pH_T_ (Fig. 1c; Theil-Sen linear regression: V = 119; df = 6; *p* = 0.2472). One of the nominal pH 7.6 and all of the nominal pH 7.35 and 7.1 cultures were excluded from the analysis due to high mortality and low number of observations. Despite growth rate was unaffected by pH larvae grown in more acidic conditions were generally smaller both during the initial D-shell phase and at day 24 (Fig. S1, supplementary material).

**Figure 3:**
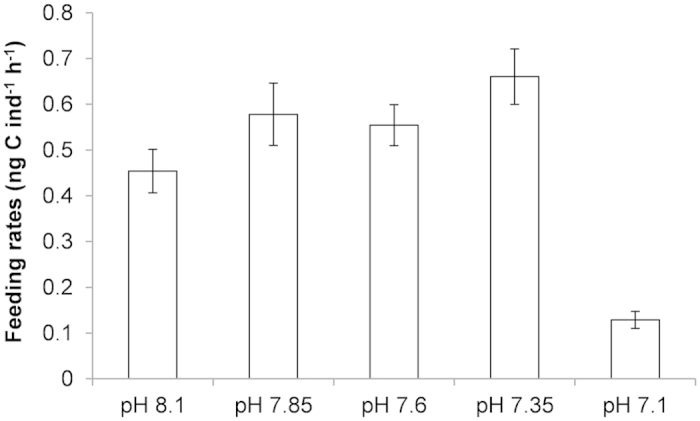
Lower feeding rates at nominal pH 7.1. Mean % ± s.e.m. feeding rates (ng C ind^−1 ^h^−1^) in *M. edulis* D-shaped larvae cultured at 5 different nominal pHs.

Larvae were feeding in all cultures (Fig. 3). Feeding rates (ng C ind^−1^ h^−1^) were significantly 4.4 times lower at the lowest nominal pH treatment (ANOVA 2, model: F_11,60_ = 3.98; *p* < 0.0001; pH: F = 10.15; *p* < 0.0001), with no difference between replicates (F = 1.83; *p* = 0.17) and no interaction between pH and replicate (F = 0.44; *p* = 0.35).

**Figure 4:**
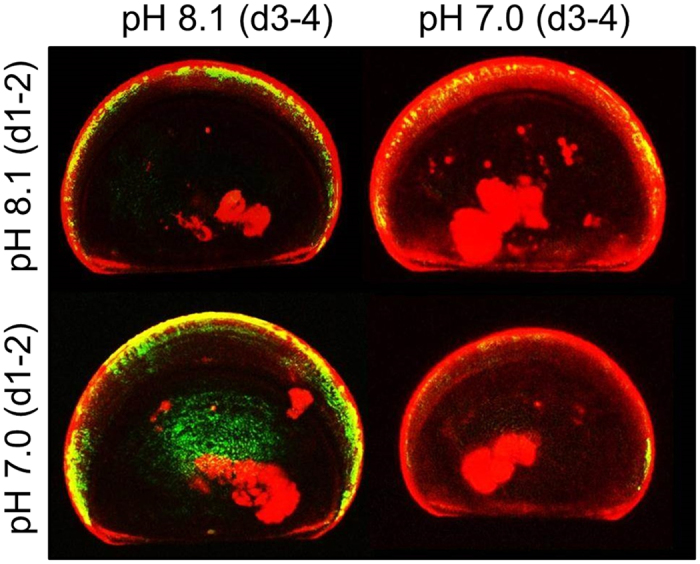
Quantification of calcification and dissolution using calcein. Confocal image of calcein-labelled skeleton of 11 day old D-shaped larvae of *M. edulis* cultured for 2 days in green calcein (d1-2, green signal) at either pH 8.1 or pH 7.0 and then transferred for 2 days in blue calcein (d3-4, red signal). Larvae exposed to pH 7.0 at days 3–4 have a larger red band revealing increased calcification as well as less green signal suggesting higher dissolution.

**Figure 5:**
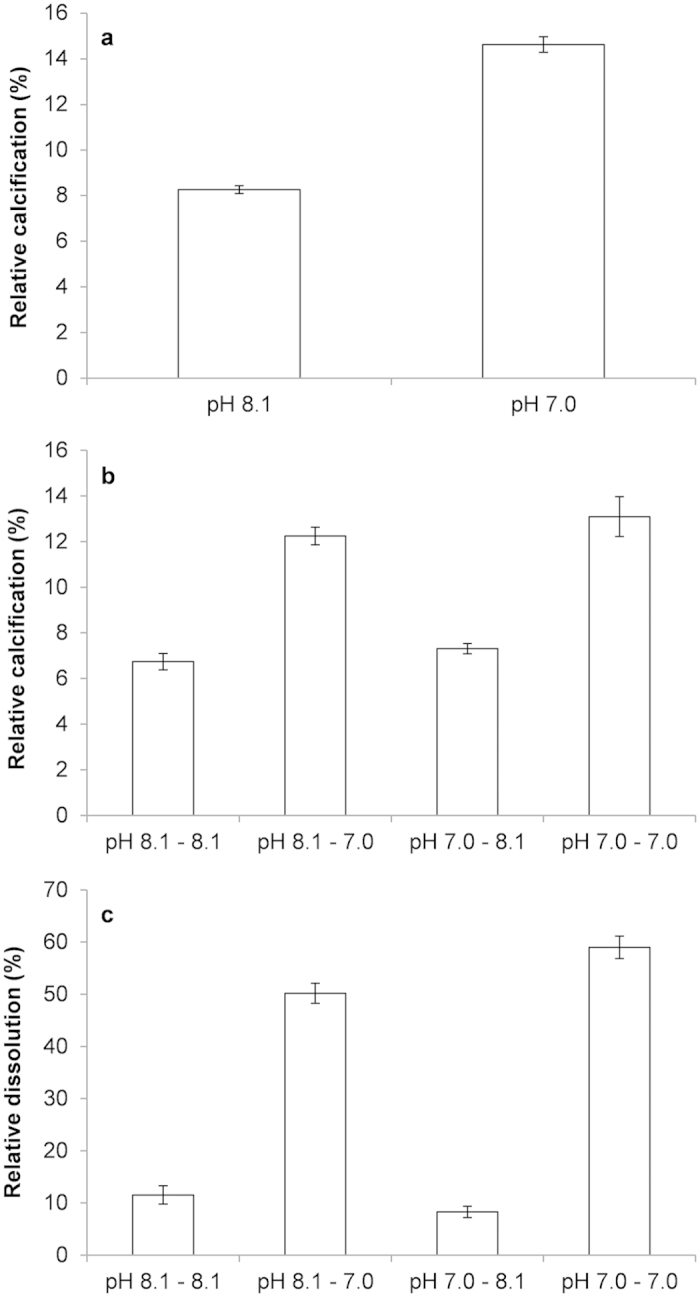
Higher calcification and shell dissolution at lower pH. Mean % ± s.e.m. relative calcification (%; (**a**,**b**)) and dissolution (%, (**c**)) in *M. edulis* 11 days old D-shaped larvae exposed to a 2 days treatment at pH 8.1 or pH 7.0 (**a**), then transferred to either the same (pH 8.1–8.1 and pH 7.0–7.0) or the other (pH 8.1–7.0 and pH 7.0–8.1) for 2 more days (**b**,**c**).

A side experiment was performed to estimate the impact of an acute exposure to pH 8.1 or pH 7.0 on relative calcification and shell dissolution of larvae raised in nominal pH 8.1 (Fig. 4). The relative calcification was 1.8 times higher when larvae were kept at pH 7.0 as compared to pH 8.1. For days 1–2, there was a significant effect of the acute pH treatment (Fig. 5a; ANOVA: F_1,10_ = 279.72, *p* < 0.0001). For days 3–4, a similar significant effect of the acute pH treatment was observed (Fig. 5b; ANOVA 2, model: F_3,20_ = 41.02; *p* < 0.0001; pH_2_: F = 121.47; *p* < 0.0001) but there was no significant effect of the pH experienced during days 1–2 (pH_1_: F = 1.40; *p* = 0.25). Dissolution was also 5 times significantly higher when larvae were exposed to the acute pH 7.0 treatment (Fig. 5c; ANOVA 2, model: F_3,20_ = 214.51; *p* < 0.0001; pH_2_: F = 629.76; *p* < 0.0001) but there was no significant effect of the pH experienced during days 1–2 (pH_1_: F = 2.48; *p* = 0.13).

## Discussion

Our results showed a relationship between decreasing pH and mussel larvae fitness related parameters including survival and percentage of shell abnormality. Moreover, larvae grown under the lowest pH (average pH_T_ = 7.16) were virtually not able to reach the shelled veliger stage. Once this acidity level was reached feeding rates also dropped significantly, which is a result of arrested development at the non-feeding trochophore or ciliated embryo stage^16^. Some of these larvae may have reached a late trochophore stage, started developing a velum and were able to consume small amounts of food, which may explain the positive feeding rate reported. Based on our results concerning shell production, feeding and development we estimated that larvae grown at average pH 7.16 were likely beyond their physiological tipping point.

Responses of blue mussel larvae to OA have previously been investigated. However previous studies have often not gone beyond end of century projected scenarios. Gazeau *et al.*^13^ report a negative effect on both hatching rates and D-veliger size of *M. edulis* larvae at pH ≈ 7.6 in comparison with control conditions (pH ≈ 8.1). However their results also support the fact mussel larvae are capable of developing a shell in seawater undersatured with respect to aragonite, which is in agreement with our findings. Very similar conclusions were reached by Bechmann *et al.*^14^ who also tested the effects of an end of century OA scenario (pH 7.6) and reported mussel larvae were still able to produce a shell in aragonite undersatured seawater but were significantly smaller than in control conditions (pH 8.1).

The overall physiological tipping point we identified is likely below present and future pH range at our sampling site (minimum pH 7.3 by 2100^15^). However, lower pH values are documented in other habitats. As an example it has been shown that along the eastern coast of the United States, eutrophic estuaries, where microbial degradation of organic matter leads to high production of CO_2_ which in turn causes an increase in seawater acidity, can be temporarily characterized by highly hypoxic waters and concomitantly high levels of *p*CO_2_ (>3000 μatm) and pH < 7.0^17^. However, results from our study cannot be extrapolated to organisms in these habitats as they may be adapted to these more extreme environments. Nevertheless our work identified previously undescribed tolerance thresholds for blue mussel larvae and goes beyond the simplistic approach comparing present average against future projected scenarios and not accounting for natural and often considerable fluctuations in seawater acidity. It has been shown that the pH_T_ in the Gullmar Fjord (Sweden), where our experimental specimens were collected, can vary between 8.7 and 7.6 throughout the years and monthly variability can reach 0.9 pH units^15^. Accounting for natural pH fluctuations is particularly important when investigating the impacts of OA on organisms living in biogeochemically complex environments such as coastal ecosystems characterized by large spatio-temporal variability in seawater pH^18^. Calcifying organisms living in coastal environments for instance, can evolve mechanisms to maintain homeostasis for calcification in low pH waters. For example, during summer upwelling events that bring more acidic bottom layer water up to the surface, pH within the inner Kiel Fjord (Germany) decreases considerably reaching values of 7.3^19^. This environment is however dominated by *M. edulis* mussels which are capable of tolerating low pH conditions^19^. It is hence necessary, as already stressed by other authors^15^, to understand and account for the characteristics of the niche occupied by the investigated species when analysing its response to environmental stressors. This is particularly important when focus is on tolerance thresholds and tipping points beyond which an organism cannot function efficiently and survive. This should be considered in a multidimensional environment including multiple drivers. As an example it has been shown that calcification of blue mussels declines linearly with increasing *p*CO_2 _11. However if food supplies are abundant juvenile mussels are capable of calcifying at high rates suggesting that mussel calcification, at least to a certain seawater acidity threshold, is mainly an energy-limited process and is still possible even at considerably low pH^19^. This is in accordance with findings by Thomsen and Melzner^12^ who suggest that the decreased shell growth in adult blue mussels exposed to severe acidification is likely to be caused by increased cellular energy demand in combination with nitrogen loss. Similar patterns have been described for other bivalve species such as the eastern oyster *Crassostrea virginica* (Gmelin, 1791) that increases its metabolic rate when exposed to high CO_2_ concentrations as a result of higher homeostasis energetic demands^20^.

Our results also confirm remarkable tolerance of *M. edulis* larvae to increased seawater acidity. Although mortality and abnormality rates increased with decreasing pH with a tipping point for normal shell growth identified at pH = 7.765, a considerable number of larvae grown at nominal pH ≥ 7.6, even in aragonite under-saturated waters, were able to feed, calcify and reach the normal veliger D-shape stage. Additionally, growth rates of normally developing larvae were not affected by pH. Ries *et al.*^21^ analysed the impact of high *p*CO_2_ on 18 species of marine calcifiers and found a decrease in calcification under more acidic conditions for several of these. However even at the highest *p*CO_2_ treatment (average *p*CO_2_ 2856 ± 54 ppm), at which seawater was under-saturated with respect to aragonite (average Ω_*a*_ 0.7 ± 0.2), adult *M. edulis* were the only organism for which net calcification was unaffected by increased seawater acidity. This is likely - at least to some extent - a result of the presence of a periostracum on their shell which protects adult mussels from dissolution. Although shells of mussel larvae are more vulnerable to dissolution than adult shells it appears that some individuals are able to maintain net calcification likely through a shift in energy budget towards this process. Shifts in energy budget in larvae exposed to ocean acidification were observed in other species. For instance, scope for growth was reduced in sea urchin larvae^15^,^22^ as a consequence of increased costs for maintenance^6^,^23^ including acid-base regulation^24^,^25^. This led to reduced growth and developmental delays. Our results suggest increased costs for net calcification. When larvae were exposed to a low pH challenge, net calcification was maintained through a dynamic process involving increased calcification to compensate for increased dissolution. Despite this increased energy cost and no significant effects on energy acquisition (feeding), growth was maintained at lower pH. It is then likely that energy was diverted from other physiological processes (e.g. energy storage) to maintain development. Such a strategy can have serious consequences for larval fitness (e.g. increased probability of mortality and abnormality) but also later developmental stages.

As previously mentioned throughout our experiment we observed an increase in larval abnormality rate with decreasing pH_T_. However, normal and abnormal D-shaped larvae were observed in most cultures and some larvae experienced arrested development at the trochophore stage even at higher pH treatments. These results reflect variability in individual responses to OA within the same population and even within the same brood of *M. edulis*. In order to clearly test the effect of pH on physiological responses of mussel larvae and reduce chances of confounding effects linked to differences in the genetic pool between replicates, our experiment was designed to minimize genetic variability by using a single set of two parents. However the observed variability in individual responses we describe may still be linked to genetic variation (e. g. recombination) or the result of differences in within-brood egg quality leading to differences in fitness and performance during the larval stage. Marshall *et al.*^26^ modelled fitness of marine invertebrates’ females which either produce equally sized offspring or offspring of variable size. They found that in unpredictably variable environments females producing offspring of variable size within each brood have higher mean fitness within generations and less variable fitness across generations. Authors also showed that within-brood offspring size variation was highest for indirect developers with feeding larvae, such as blue mussels. Although it is known *M. edulis* generally produces large numbers of relatively small yolk-poor eggs^27^, the variability we observed may be the result of within-brood egg size/quality variability and under more stressful conditions could increase the probability of at least some offspring successfully completing larval development and recruiting into the adult population.

To summarize, *M. edulis* larvae show a considerable degree of tolerance to OA. We identified a tipping point for normal shell development corresponding to pH_T_ = 7.765 and concluded that larvae grown at average pH 7.16 were beyond their physiological tolerance threshold. Under milder acidification larval developmental patterns suggest great variability in individual responses to OA even within the same larval brood which may be a result of genetic or egg/size quality variability and may increase resilience of mussel populations in environmentally heterogeneous environments such as shallow coastal waters. Additionally we observed no impact of pH on growth rates of normally developing larvae, a likely consequence of shifts in energy budget toward calcification which resulted in unaffected net calcification. These results should not be extrapolated to different *M. edulis* populations or the entire species as this was not the objective of our study. Our goal was to test physiological tolerance in a species considered susceptible to OA and stress the importance of potential local adaptation. Further work rigorously testing for adaptation potential to more acidic conditions is needed in order to determine the true extent of the threat posed by OA for blue mussel populations.

## Materials and Methods

### Broodstock collection and larval rearing

*M. edulis* broodstock specimens (n = 22, mean shell length size 7.94 cm ± 0.08) were collected from Blåbärsholmen (58° 14′ 53.55″ N–11° 26′ 16.84″ E, Gullmar fjord, Sweden) on 30^th^ April 2014, brought back to Sven Lovén Centre for Marine Sciences – Kristineberg (Fiskebäckskil, Sweden) and kept in a flow through system (temperature 9.14 °C ± 0.03; pH_T_ ≈ 8.1; salinity 33.69 ± 0.01). After 41 days, spawning was induced by rapid increase of filtered sea water (FSW) temperature (+10 °C). Active sperm from one male was added to a 250 ml solution of FSW and eggs from one female at ambient temperature. We minimized the number of parents in order to have replicates containing larvae with a similar genetic background which allows clearly linking physiological responses to the environmental parameter tested. Despite not allowing extrapolations of our results to a wider population this approach reduces chances of confounding effects linked to differences in the genetic pool between replicates (see Cornwall and Hurd^28^ for recent controversy on this issue). Fertilization success was assessed by the release of polar bodies. Developing embryos were added to cultures 3 hours post-fertilization at a density of ≈16 embryos ml^−1^. Each culture consisted in 5000 ml flasks filled with aerated FSW, previously equilibrated to the target pH and placed in a temperature controlled room (9 °C). Seawater was constantly aerated and mixed through air bubbling. Cultures were diluted after 3 days to reach a density of ≈10 embryos ml^−1^. Larvae were fed daily with the microalgae *Rhodomonas sp.* from day 5 onwards. In order to guarantee equal amounts of energy resources in each culture algae concentrations were adjusted daily to 150 μg C l^−1^ after calculations of algal size and density using a Coulter counter (Elzone 5380; Micrometrics) and equivalent carbon content^29^.

### Experimental design and carbonate chemistry

Larvae were exposed in triplicate for 48 days to 5 different pHs (randomised design): nominal pH 8.1 (control pH), 7.85, 7.6, 7.35 and 7.1. In order to reach the target pH pure CO_2_ was bubbled into each culture and pH controlled through a pH-stat system (Aqua Medic, Bissendorf, Germany). Every second day, after calibration using TRIS (Tris/HCl) and AMP (2-aminopyridine/HCl) buffer solutions with a salinity of 32, pH was measured on the total scale (pH_T_). Total alkalinity (TA) was measured every second day from filtered water samples (0.45 μm) and carbonate system parameters were calculated from pH_T_ and TA using CO2SYS (Lewis & Wallace, 1998).

### Mortality, growth and abnormality rate

After being killed using one drop of paraformaldehyde solution (4% PFA in FSW, buffered at pH 8.3), larvae were counted in two 10 ml subsamples for each replicated flask daily up to day 15, once every other day up to day 29 and finally on days 35, 38, 41 and 48. On average 17 larvae per sampling point and from each culture were photographed for further measurements. Determination of larval abnormalities was based on His *et al.*^30^. Shell length was measured using the software ImageJ (Abràmoff *et al.*) as maximum length along the anterior/posterior axis parallel to the hinge.

### Feeding rate

Feeding rates were determined using larvae subsampled from main cultures at days 10, 14 and 16 post fertilization as described in Stumpp *et al.*^22^. Feeding rate was estimated as clearance rate by measuring the algal concentration at the end of the incubation (48 h) in control (no larvae, n = 3 per pH) and experimental flasks (20 larvae, n = 2 per replicate). Feeding trials were performed at the corresponding nominal pH of the tested replicated flask. The rates were presented as rates per larvae in ng C ind^−1 ^h^−1^.

### Relative calcification and dissolution

At 11 days post-fertilization, 20 D-shaped larvae from the nominal pH 8.1 treatments were transferred to 10 ml of seawater previously equilibrated at either pH_T_ 8.1 or 7.0 and containing 25 μg/ml green calcein (Sigma, C0875). When calcein is present in the seawater, it is incorporated into newly built skeleton. These structures are then labelled in green/yellow. After 48 h, larvae (n = 6 per treatment) were examined using a Leica confocal microscope. Relative net calcification (%) was estimated as the ratio between the length of the green labelled calcein band and the maximum length.

Larvae were then transferred to seawater equilibrated at the same pH (from 8.1 to 8.1 or 7.0 to 7.0) or the other pH (from 8.1 to 7.0 or 7.0 to 8.1) in a fully crossed design and containing 25 μg/ml blue calcein (Sigma, M1255). Larvae (n = 6 per treatment) were examined using a Leica confocal microscope. Relative net calcification (%) was estimated as the ratio between the size of the blue labelled calcein band and the maximum length. Relative dissolution (%) was estimated as:


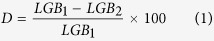


where *D* = dissolution (%); *LGB*_1_ = length of the green band after a two day exposure; *LGB*_2_ = length of the green band after a four day exposure.

### Data analysis

Each mean value is expressed with its standard error of mean (mean ± s.e.m.). Statistical analyses were conducted using R (R Development Core Team, R: http://www.R.org/. 2011) and SAS (SAS Institute 1990). When parametric statistical tests were employed data was checked for homoscedasticity and normal distribution.

Larval mortality rates (ln (relative density) ln (day)^−1^) corresponded to the slope coefficients derived from power regressions (Table S1, supplementary material) with the following general equation:


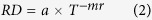


where *RD* = relative density (relative to initial density); *a* = intercept; *T* = time (days); *mr* = mortality rate.

The effect of pH_T_ on mortality rates was then analysed by simple linear regression.

In order to investigate normal versus abnormal larval development larvae were categorized as arrested development (trochophores or ciliated embryos), abnormally D-shaped and normally D-shaped (Fig. 2). The relationship between pH_T_ and abnormality (% of abnormally D-shaped larvae + arrested development) was investigated using the following Gompertz equation:


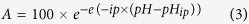


where *A* = abnormality (%); ip = inflexion point; *pH*_*ip*_ = pH at *ip* (abnormality tipping point). Maximum % abnormality was set at 100. Mean abnormality percentage was calculated for each replicate within each treatment from day 4 (when most larvae had reached the D-shape stage) up to day 48. For some cultures due to limitations in sample sizes abnormality percentages could not be calculated up to day 48 (Table S2, supplementary material).

Growth rates were calculated from day 4 (start of D-shape for most replicates) up to day 24 as the slope coefficients derived from Theil-Sen median based non-parametric linear models with time (day) as the independent variable and shell length as the dependent (Table S3, supplementary material). The effect of pH_T_ on growth rates of normally D-shaped larvae was then analysed using a Theil-Sen median based linear regression. Theil-Sen regressions were used as data did not meet assumptions for linear regressions.

Impacts of nominal pH and replicates on feeding rates were tested with a 2-way ANOVA model. Impact of experimental pHs on relative calcification and dissolution rates was tested using 1- and 2-way ANOVA.

## Additional Information

**How to cite this article**: Ventura, A. *et al.* Maintained larval growth in mussel larvae exposed to acidified under-saturated seawater. *Sci. Rep.* **6**, 23728; doi: 10.1038/srep23728 (2016).

## Supplementary Material

Supplementary Information

## Figures and Tables

**Table 1:** Mean ± s.e.m. of seawater carbonate chemistry parameters for each culture.

Nominal pH	Measured			Calculated		
	pH_T_	T (°C)	TA (mmol kg^−1^)	*p*CO_2_ (μatm)	Ω_*a*_	Ω_*c*_
8.1	8.12 ± 0.007	9.20 ± 0.14	2.3 ± 0.009	334 ± 6	2.35 ± 0.03	3.71 ± 0.06
8.1	8.12 ± 0.008	9.19 ± 0.15	2.3 ± 0.010	335 ± 8	2.30 ± 0.04	3.63 ± 0.06
8.1	8.11 ± 0.006	9.28 ± 0.13	2.3 ± 0.012	350 ± 6	2.29 ± 0.02	3.62 ± 0.04
7.85	7.87 ± 0.018	10.64 ± 0.23	2.4 ± 0.039	675 ± 35	1.58 ± 0.05	2.49 ± 0.09
7.85	7.86 ± 0.017	9.45 ± 0.09	2.4 ± 0.021	683 ± 28	1.45 ± 0.05	2.29 ± 0.09
7.85	7.80 ± 0.031	10.06 ± 0.14	2.3 ± 0.010	693 ± 48	1.46 ± 0.09	2.31 ± 0.16
7.6	7.67 ± 0.020	9.36 ± 0.09	2.4 ± 0.015	1093 ± 49	0.96 ± 0.04	1.51 ± 0.07
7.6	7.64 ± 0.021	9.43 ± 0.12	2.3 ± 0.009	1139 ± 39	0.88 ± 0.03	1.39 ± 0.05
7.6	7.57 ± 0.022	9.42 ± 0.11	2.3 ± 0.010	1352 ± 78	0.75 ± 0.03	1.19 ± 0.05
7.35	7.46 ± 0.051	9.46 ± 0.11	2.3 ± 0.011	1860 ± 150	0.63 ± 0.06	0.99 ± 0.01
7.35	7.41 ± 0.013	9.75 ± 0.11	2.3 ± 0.010	1997 ± 66	0.54 ± 0.01	0.86 ± 0.02
7.35	7.41 ± 0.017	9.55 ± 0.14	2.4 ± 0.012	2012 ± 83	0.55 ± 0.02	0.87 ± 0.03
7.1	7.17 ± 0.024	9.81 ± 0.14	2.4 ± 0.015	3605 ± 167	0.32 ± 0.01	0.51 ± 0.02
7.1	7.17 ± 0.038	9.38 ± 0.12	2.4 ± 0.012	3656 ± 287	0.32 ± 0.02	0.51 ± 0.03
7.1	7.15 ± 0.021	9.54 ± 0.12	2.3 ± 0.015	3712 ± 160	0.30 ± 0.01	0.48 ± 0.01

Measured pH on the total scale (pH_T_), temperature (T; °C) and total alkalinity (TA; mmol kg^1^) were used to calculate CO_2_ partial pressure (*p*CO_2_; μatm), aragonite and calcite saturation states (Ω_*a*_ and Ω_*c*_, respectively).

**Table 2:** Mean % ± s.e.m. normally D-shaped, abnormally D-shaped and trochophore larvae in treatments and control.

Nominal pH	Normally D-shaped		Abnormally D-shaped		Trochophores	
	Mean (%)	± s.e.m.	Mean (%)	± s.e.m.	Mean (%)	± s.e.m.
8.1	84.01	1.80	14.20	3.31	0.20	0.08
7.85	83.77	3.22	15.76	3.28	0.46	0.26
7.6	42.06	18.06	27.39	11.17	30.54	24.00
7.35	6.06	4.37	59.92	24.01	34.00	26.78
7.1	1.39	0.52	1.37	0.62	97.22	0.46

Means calculated on mean % abnormally D-shaped larvae from replicates within each nominal pH treatment.
