# Corrigendum: Maintained larval growth in mussel larvae exposed to acidified under-saturated seawater

**DOI:** 10.1038/srep46999

**Published:** 2018-06-27

**Authors:** Alexander Ventura, Sabrina Schulz, Sam Dupont

Scientific Reports 6; Article number: 2372810.1038/srep23728; published online: 03 29 2016; updated: 06 27 2018

This Article contains errors.

In Table 1, in the sixth row, the pH_T_ value for nominal pH 7.85,

“7.80 ± 0.031”

should read:

“7.86 ± 0.031”

Additionally, the legend for Figure 2,

“(**a**) Trochophore stage, (**b**) protruding mantle, (**c**) indented margin, (**d**) convex hinge, (**e**) cupped and (**f**) normally D-shaped larva. N. B. All photos are from experiment day 11.”

should read:

“(**a**) Trochophore stage, (**b**) protruding mantle, (**c**) indented margin, (**d**) convex hinge, (**e**) cupped and (**f**) normally D-shaped larva. N. B. The larva in panel (**a**) is from experiment day 4 whilst larvae in remaining panels are from day 11”.

