# Supplementary material for: Maintained larval growth in mussel larvae exposed to acidified under-saturated seawater
Source: Sci Rep. 2016 Mar 29;6:23728. doi: 10.1038/srep23728 (PMC4810423; doi:10.1038/srep23728)
Supplement: Supplementary Information [file srep23728-s1.pdf]

Maintained larval growth in mussel larvae exposed to acidified under-saturated seawater

Alexander Ventura**, Sabrina Schulz and Sam Dupont

Department of Biological and Environmental Sciences, University of Gothenburg, The Sven Lovén Centre for Marine Sciences - Kristineberg 566, SE-451 78 Fiskebäckskil, Sweden

*Corresponding author: alexander.ventura@bioenv.gu.se

SUPPLEMENTARY MATERIAL

Table S1. Larval mortality rates (ln (relative density) ln $(day)^{-1}$) were calculated as the coefficients of the significant power regressions between relative density and time. Results of the regressions (Intercept, *p*-value, R^2, F-value and df: degrees of freedom) are given for each culture replicate with corresponding average pH (pH_T)

Nominal pH	Average pH_T	Mortality rate	Intercept	*p-value*	R^2	*F-value*	*df*
8.1	8.12	-0.5615	0.3589	1.24e-11	0.869	152.6	23
8.1	8.12	-0.911	0.9306	8.85e-07	0.6575	44.1	23
8.1	8.11	-0.8261	0.6943	1.99e-07	0.6985	53.2	23
7.85	7.87	-0.739	0.3805	1.78e-12	0.8892	184.6	23
7.85	7.86	-0.7066	0.5166	6.59e-10	0.8154	101.6	23
7.85	7.86	-0.8837	0.8549	1.38e-10	0.8387	119.6	23
7.6	7.67	-0.9298	0.9513	4.68e-10	0.8344	110.9	22
7.6	7.64	-1.5882	1.3783	1.31e-05	0.7287	40.2	15
7.6	7.57	-0.5909	0.3372	1.35e-14	0.9274	293.8	23
7.35	7.46	-1.6526	1.2511	2.88e-07	0.8155	70.7	16
7.35	7.41	-1.1152	1.0177	2.66e-08	0.7779	73.5	21
7.35	7.41	-1.4909	1.8514	1.65e-07	0.7196	56.4	22
7.1	7.17	-1.6691	1.3608	4.61e-08	0.835	86	17
7.1	7.17	-1.5979	1.1704	2.17e-07	0.8218	73.8	16
7.1	7.15	-1.2467	1.1464	7.45e-05	0.6355	27.8	16

Table S2. Number of days used for calculation of percentage abnormality for each replicate. Corresponding average pH (pH_T) for each replicate is reported.

Nominal pH	pH_T (mean)	Days
8.1	8.100	24
8.1	8.126	48
8.1	8.118	41
7.85	7.878	48
7.85	7.860	48
7.85	7.863	48
7.6	7.575	48
7.6	7.672	48
7.6	7.610	15
7.35	7.391	24
7.35	7.413	24
7.35	7.502	17
7.1	7.147	15
7.1	7.152	20
7.1	7.114	15

Table S3. Growth rates (μm day^{-1}) were calculated as the coefficients of the significant Theil-Sen median based linear regressions. Results of the regressions (Intercept, *p*-value, MAD, Residual s. e. m., V value and df: degrees of freedom) are given for each culture replicate with corresponding average pH (pH_T). One replicate from nominal pH 7.35 and two from nominal pH 7.1 not included due to too low number of observations.

Nominal pH	Average pH_T	Growth rate	Intercept	*p-value*	MAD	Residual SE	*V value*	*df*
8.1	8.11	2.000	102.000	<2e-16	1.853	9.163	449615226	266
8.1	8.10	2.188	101.875	<2e-16	1.761	9.057	544424732	277
8.1	8.11	1.091	110.091	<2e-16	1.718	7.843	302796263	247
7.85	7.88	2.375	99.000	<2e-16	1.709	8.538	495044827	267
7.85	7.85	2.000	100.000	<2e-16	1.853	8.672	266793543	234
7.85	7.82	2.286	97.571	<2e-16	1.906	8.842	520185301	273
7.6	7.66	2.000	87.000	<2e-16	2.372	11.800	13364948	111
7.6	7.59	2.400	90.400	<2e-16	1.949	9.369	334689355	244
7.6	7.64	-0.500	111.500	0.7500	2.224	7.071	2	2
7.35	7.39	3.190	54.430	0.00932	1.831	14.140	241.5	6
7.35	7.38	2.222	70.889	1.19e-07	4.989	24.110	32662	25
7.1	7.15	-4.250	142.125	0.0625	6.005	13.98	0	2

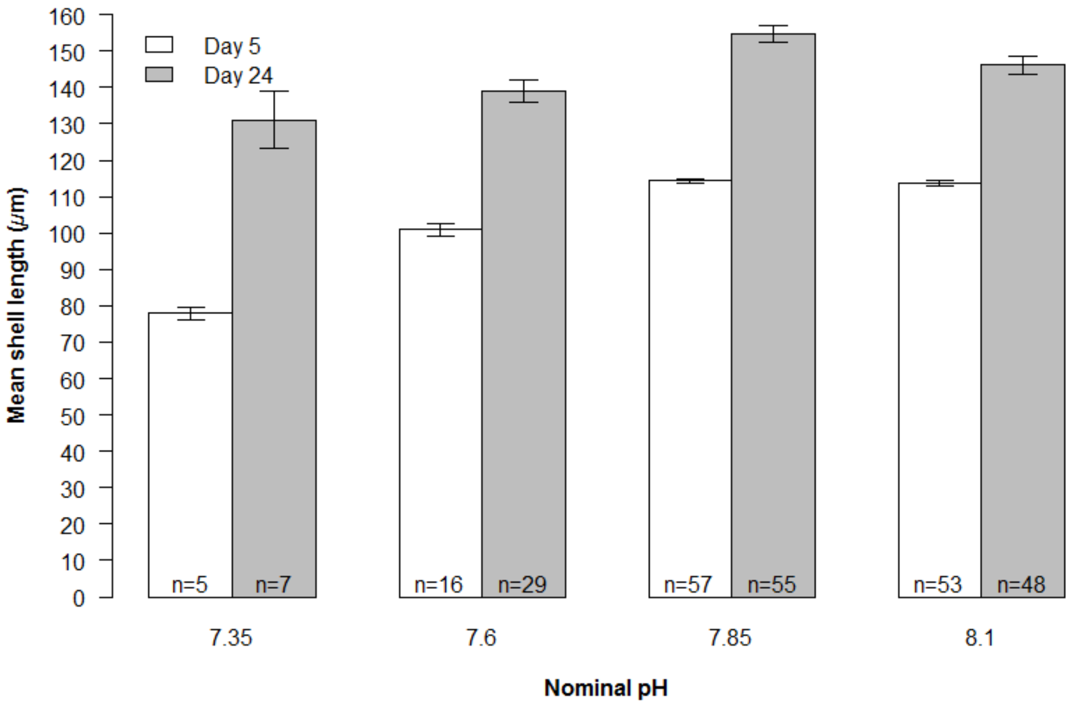

Fig S1 | Increasing shell length with increasing pH. Mean ± s. e. m. of larval shell length at day 5 (white bars) and day 24 (grey bars) at different nominal pHs. N = number of observations
